# Increased bone resorption by long-term cigarette smoke exposure in animal model

**DOI:** 10.1016/j.heliyon.2021.e08587

**Published:** 2021-12-10

**Authors:** Jader Joel Machado Junqueira, Juliana Dias Lourenço, Kaique Rodrigues da Silva, Vanda Jorgetti, Rodolfo P. Vieira, Amanda Aparecida de Araujo, Kátia De Angelis, Aristides Tadeu Correia, Luan Henrique Vasconcelos Alves, Iolanda de Fátima Lopes Calvo Tibério, Alexandre Póvoa Barbosa, Fernanda Degobbi Tenorio Quirino dos Santos Lopes

**Affiliations:** a Department of Medicine, Laboratory of Experimental Therapeutics (LIM-20), School of Medicine, University of São Paulo, São Paulo, SP, Brazil; b Department of Medicine, Laboratory of Renal Physiopathology (LIM-16), School of Medicine, University of São Paulo, São Paulo, SP, Brazil; c Post-graduation Program in Sciences of Human Movement and Rehabilitation, Federal University of São Paulo (UNIFESP), Santos, SP, Brazil; d Post-graduation Program in Bioengineering, Brasil University, São Paulo, SP, Brazil; e Brazilian Institute of Teaching and Research in Pulmonary and Exercise Immunology (IBEPIPE), São José dos Campos, SP, Brazil; f Exercise Physiology Laboratory, Federal University of São Paulo (UNIFESP), São Paulo, SP, Brazil

**Keywords:** Smoking, Bone remodeling, Bone matrix, Oxidative stress

## Abstract

**Introduction:**

Clinical and experimental studies have been attesting the deleterious effects of smoking mainly due to the stimulation of osteoclastogenesis and inhibition of osteoblastogenesis. However the physiological mechanisms that can explain these changes are not fully understood.

**Aims:**

To evaluate the trabecular bone resorption effect caused by long-term exposure to cigarette smoke and the action of cytokines and reactive oxygen species involved in this process.

**Methods:**

Sixty young adult C57BL/6 mice were allocated to two groups: control, 30 animals exposed to filtered air for 1, 3 and 6 months; and smoke, 30 animals exposed to cigarette smoke for 1, 3 and 6 months. Femoral and tibial extraction was performed to evaluate the bone mineral matrix, bone cytokines (Receptor activator of nuclear factor-kappa B ligand - RANKL and Osteoprotegerin - OPG) and oxidative stress markers (Thiobarbituric acid reactive substances - Tbars).

**Results:**

Exposure to cigarette smoke (CS) generated changes in bone structural parameters in the 6th month of follow-up, demonstrating an evident bone loss; reduction in OPG/RANKL ratio from the 3rd month on and increase in Tbars in the first month, both closely related to the increase in osteoclastogenic activity and bone resorption.

**Conclusion:**

These findings reinforce the importance of CS-induced oxidative stress in bone compromising the bone cellular activities with a consequent impairment in bone turn over and changes in bone structure.

## 1 Introduction

Cigarette smoke is recognized by their toxic and carcinogenic actions in human health [1, 2]. In bone, clinical and experimental studies have been attesting the deleterious effects of smoking [3, 4].

Since the 70s, the association between smoking and fractures due to bone insufficiency in postmenopausal women had already been described [5]. Experimental and *in vitro* studies have also shown that smoking has the potential to act on bone remodeling, inducing bone loss and reducing bone mineral density [6, 7, 8]. These effects are mainly due to the stimulation of osteoclastogenesis and inhibition of osteoblastogenesis [6, 9].

Bone is an organized and specialized connective tissue composed of cells and a calcified extracellular matrix [10]. The extracellular matrix is responsible for conferring structural properties on bone, as well as assisting in some cellular regulatory functions. Its organic portion includes collagen fibers, proteoglycans, matrix proteins, cytokines (e.g. RANKL and OPG) and growth factors [11]. There are two large groups of specialized cells in bone tissue that make up the so-called multicellular bone unit and that are closely related to bone remodeling: osteoblastic lineage cells (osteoblasts, osteocytes and lining cells or lining-cells) and bone resorption cells (osteoclasts). Considering that bone remodeling is a continuous process of bone renewal that depends on the coordinated cellular activity, it is important to better understand how the different bone cells as well as different chemokines interfere in bone formation and bone resorption [10].

The receptor activator of nuclear factor-kappa B ligand (RANKL or CD254), a member of the superfamily of tumor necrosis factors (TNF), is produced by osteoblasts during the resorption phase of bone remodeling. It has been described as an inflammatory marker of bone resorption as it stimulates osteoclast proliferation, differentiation, activation and survival through RANK-RANKL binding [12, 13]. Osteoprotegerins (OPG), also produced by osteoblasts, are natural inhibitors of the action of RANKL, as they prevent the RANKL-RANK binding from occurring, thus preventing osteoclast differentiation and action [14]. Thus, reductions in its expression level would no longer inhibit bone resorption. The influence of smoking and pollution on the RANKL/OPG ratio has been recently demonstrated, and this relationship is one of the main determinants of bone mass [12, 14]. These processes allow the bone to have a plastic capacity to adapt to external stress (traction and compression) to which it has been submitted over the years.

However, the exposure to dangerous exogenous factors, such smoking, could interfere in bone remodelling. The chronic inflammatory process associated with smoking is also capable to promote a favorable environment for bone resorption mediated by increased production of reactive oxygen species (ROS) [15]. Deleterious effects of oxidative stress result from peroxidation of lipid membrane, protein inactivation and damage in nuclear DNA integrity, increasing the risk of mutations and leading to cellular apoptosis and necrosis [16]. In bone tissue ROS can also be produced after RANK-RANKL binding and acting as second messengers in signaling pathways involved in osteoclastogenesis [15, 17].

Although studies have shown the role of smoking in bone loss, reduction in bone mineral density, stimulation of osteoclastogenesis and inhibition of osteoblastogenesis, in our knowledge, there is no a temporal study that evaluate how these physiological events can interfere with each other in different time points during smoking exposure.

Thus, the aim of this study was to perform temporal analysis to evaluate the trabecular bone resorption effect caused by long-term exposure to cigarette smoke and the importance of ROS production in the increase of RANKL/OPG ratio that leads the osteoclastogenesis.

## 2 Methods

### 2.1 Experimental procedures

This experimental study required sixty C57BL/6 young adult male mice (6–8 weeks old), with an average weight of 26 g, provided by the Central Animal Facility of the University of São Paulo School of Medicine. All animals received care in compliance with the Guide for the Care and Use of Laboratory Animals [18]. This study was approved by the Ethical Committee for Animal Studies from the University of São Paulo School of Medicine (Comissão de Ética no Uso de Animais – CEUA – Project Number 937/17).

The animals were housed in specific polypropylene boxes for mice (approximately 5 animals/box) with the following measures (30 × 20 × 13 cm) at the Experimental Therapeutics Laboratory. All animals had unrestricted access to water and feed (Nuvilab® CR-1 Irradiada, Quimtia S.A., Colombo/PR, Brazil) containing 1.4% calcium and 0.8% phosphorus. The animals were also maintained in a 12-hour light-dark cycle, with light from 7 am to 7 pm, at an ambient temperature of 20 ± 2 °C and were cared for a trained and qualified professional. An acclimatization period of at least 3 weeks was established.

### 2.2 Experimental groups

The animals were randomly assigned (**simple randomization 1:1, using Microsoft Excel spreadsheet)** to two groups: control (C), composed of 30 animals exposed to filtered air for 1 month (C1 = 10), 3 months (C3 = 10) and 6 months (C6 = 10) until euthanasia; smoke (S), composed of 30 animals exposed to cigarette smoke for 1 month (S1 = 10), 3 months (S3 = 10) and 6 months (S6 = 10) until euthanasia.

### 2.3 Exposure to cigarette smoke

**Figure 1:**
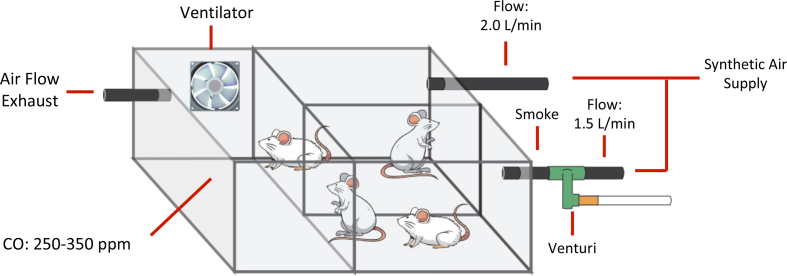
Cigarette smoke exposure apparatus.

Exposure to cigarette smoke was performed according to a protocol previously described by Toledo AC et al. [19], which involves using a 28 L inhalation chamber containing two inlet points for air and smoke supplies, one outlet point and one aerator to increase the air/smoke mixture. The synthetic air, connected to one of the inlets, has a flow rate of 2 L/min. The synthetic air connected to the second inlet has a flow rate of 1.5 L/min and passes through a Venturi system connected to a lit cigarette that suctions the smoke into the chamber (Figure 1). The levels of carbon monoxide (CO) maintained inside the chamber ranged from 250 to 350 ppm. The animals were exposed to 10 commercially filtered cigarettes per exposure (0.8 mg nicotine, 10 mg tar and 10 mg CO). This form of exposure maintains an average concentration of carboxyhemoglobin at 10 ± 1.3% and a total concentration of particulate matter at approximately 354.8 ± 50.3 μg/m^3^ day. Animals were exposed twice a day for 30 min per exposure, 5 days per week over 1, 3 and 6 months depending on the exposure group (S1 = 1 month, S3 = 3 months, S6 = 6 months). Control animals C1, C3 and C6 received only filtered air for 1, 3 and 6 months.

During the exposure to cigarette smoke, we had some animal losses related to the time of exposure and prolonged follow-up. Losses occurred in groups C6 (3 animals), S1 (1 animal) and S3 (2 animals).

### 2.4 Cell and bone mineral matrix evaluation

The tibiae of each animal were extracted and cleaned of the adjacent tissues, followed by immersion in 70% alcohol and prepared in a methyl methacrylate incorporation solution for non-descaled bone, according to the technique previously described [20]. Through a PolycutS equipped with a tungsten knife (Leica, Heidelberg, Germany) 12 histological sections of 5μm thickness were obtained, distributed in 6 slides with two cuts each and stained with 0.1% toluidine blue, pH 6.4 and coverslips with medium support Entellan H (Merck, Darmstadt, Germany).

Static, structural and dynamic parameters of bone formation and resorption were measured in 8–10 fields within the tibial proximal metaphysis using a standardized method of image analysis in which the structures of interest were marked manually using a microscope (Nikon, Labophot-2A, Japan) with 250x magnification, cursor and digitizer board. The final calculation of the parameters was performed using a specific software for histomorphometry - OsteoMeasure (OsteoMetrics, Inc., Atlanta, GA, USA).

All measurements of histomorphometric parameters were performed in the metaphyseal bone region, below the lowest point of the growth plate (below the primary spongy) and internal to the lateral cortex, excluding the cortical bone [20]. This selected area is composed of spongy bone rich in trabeculae (secondary spongy). All parameters were measured according to the recommendations of the American Society of Bone Mineral Research Histomorphometry Nomenclature Committee [21].

Histomorphometric parameters are generally divided into structural and remodeling, the latter being subdivided into resorptive and formative. **Structural parameters** include the relationship between trabecular bone volume and total bone volume (BV/TV), trabecular thickness (Tb.Th), trabecular number (Tb.N) and trabecular separation (Tb.Sp). **Remodeling parameters** that include resorptives such as the eroded surface area (ES/BS) and the osteoclastic surface (Oc.S/BS); and formative ones such as osteoid thickness (O.Th), osteoid surface area (OS/BS), osteoblastic surface (Ob.S/BS) (Table 1). All analyzes were carried out with the evaluator's blinding. The indices were all reported using the nomenclature recommended by the American Society for Bone and Mineral Research [21].

**Table 1:** Histomorphometric parameters evaluated.

Histomorphometric parameters	Abbeviation	Unit
**Structural parameters:**		
- Ratio of trabecular bone volume to total bone volume	BV/TV	%
- Trabecular thickness	Tb.Th	μm
- Trabecular number	Tb.N	mm^−1^
- Trabecular separation	Tb.Sp	μm

**Resorptive remodeling parameters:**		
- Area of eroded surface	ES/BS	%
- Osteoclastic surface	Oc.S/BS	%

**Formative remodeling parameters:**		
- Osteoid thickness	O.Th	μm
- Osteoid surface	OS/BS	%
- Osteoblastic surface	Ob.S/BS	%

### 2.5 Cytokines analysis

**Figure 2:**
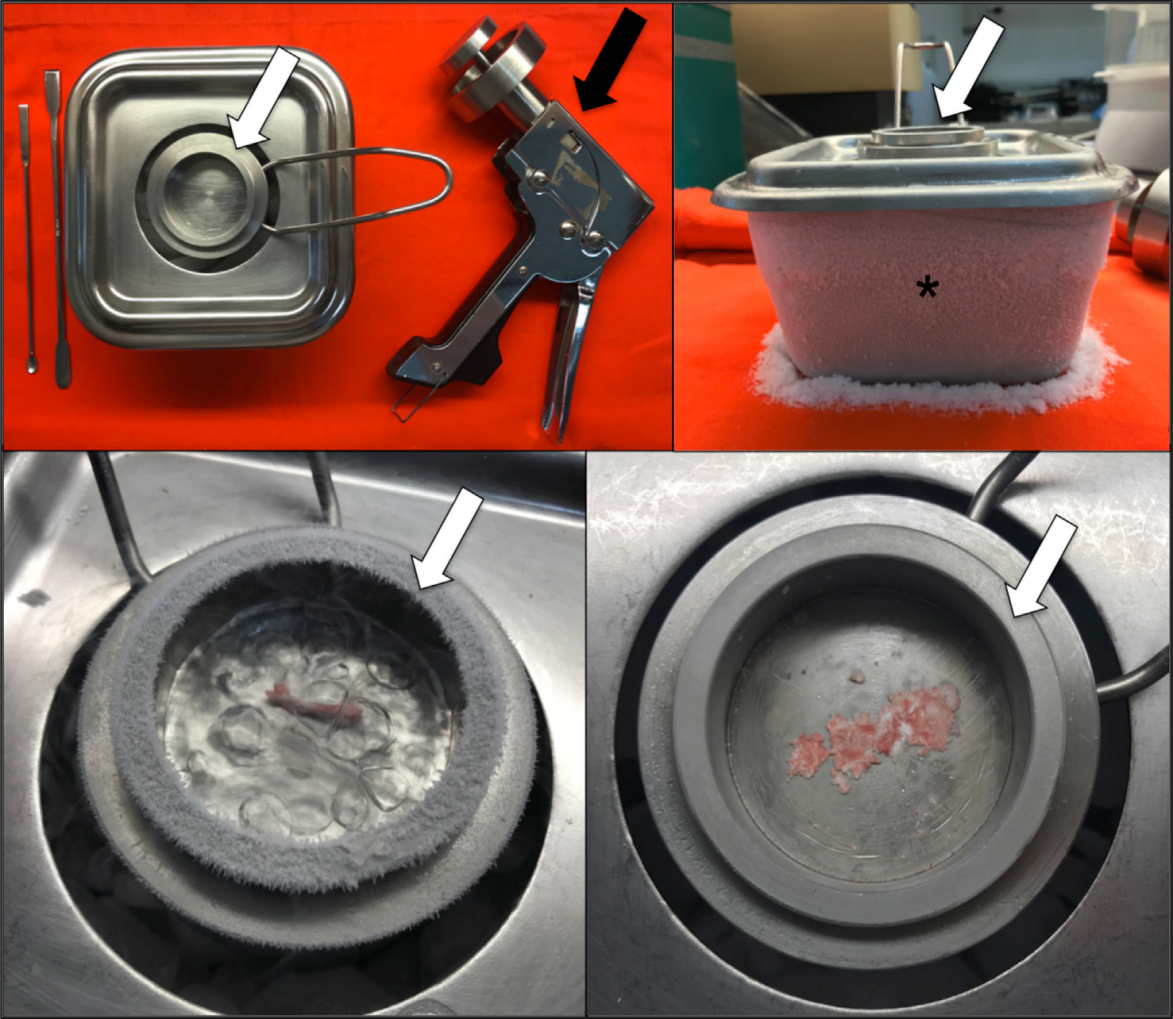
Device composed of a larger lower metallic reservoir (asterisk) where dry ice is placed and a smaller upper metallic reservoir (white arrow) where femoral bone homogenate is obtained through manual shots with a specific pistol (black arrow).

The femoral bone of each animal, previously extracted and cleaned of the adjacent tissues, were stored at -80 °C. After the end of the exposures in all groups, the tissues were homogenized and the evaluations of the expressions of RANKL and OPG were performed by an enzyme-linked immunosorbent assay method (ELISA - Enzyme Linked ImmunoSorbent Assay). Bone homogenate was obtained through the use of a specific metallic device (Figure 2) composed of a large lower reservoir containing dry ice and a small upper reservoir where the samples were placed and macerated using a manual pressure gun. Three shots were fired directly on the sample to produce the bone homogenate, which was then collected for analysis.

The analyzes were performed according to the specifications of each manufacturer:

• RANKL (Mouse TRANCE/RANK L/TNFSF11 Quantikine ELISA Kit - R&D Systems, Minneapolis, MN, USA);
• OPG (Mouse Osteoprotegerin/TNFRSF11B Quantikine ELISA Kit - R&D Systems, Minneapolis, MN, USA);

For the analysis of the samples by the ELISA method, microplates previously coated with capture antibodies were used. The samples were then added to the analysis wells so that the studied protein binds to the capture antibody. A secondary detection antibody labeled with biotin was added to the solution so that it also binds to the studied protein. A streptavidin polymer was used to label secondary detection antibodies. Subsequently, a solution containing tetramethylbenzidine substrate was added to the solution generating the appearance of a bluish color with a tonality proportional to the amount of the studied protein. The reaction was interrupted generating a change in the color of the solution to yellow and then subjected to color absorbance analysis.

### 2.6 Oxidative stress analysis

For the reaction, it was added to 150μl of plasma, 150μl of Sodium Dodecyl Sulfate (SDS) at 8.1% (W/V), 300μl of Trichloroacetic Acid (TCA) (Vetec Quimica Fina Ltda.) at 20%(P)/V) and 500μl of Thiobarbituric Acid (Tbars) (Sigma-Aldrich Corporation). This mixture was incubated for 20–30 min at 95 °C, forming a pink compound and then cooled on ice. After this procedure the tubes were centrifuged at a speed of 4000 rpm for 5 min (Eppendorf AG, Germany); 200ul of the supernatant was removed and added to an Elisa plate well. The reading was taken at 535nm in an Elisa Plate reader (Robonik, India) [22].

Proteins were quantified using the method described by Lowry et al. (1951), which uses a bovine albumin solution at a concentration of 1 mg/mL as a standard [23].

### 2.7 Statistical analysis

All comparison data among the smoke (S) and control (C) at the different times studied were performed using IBM SPSS® Statistics V21.0 for Windows software. The level of significance used was 5% (α = 0.05).

The descriptive analyses for the quantitative data that showed normal distribution are expressed as the mean and standard deviation. For quantitative data without a normal distribution, the results are expressed as the median and interquartile range IQ (25–75%). The assumptions of the normal distribution in each group and the homogeneity of the variances among groups were evaluated using the Shapiro-Wilk test and the Levene test, respectively.

For the quantitative data that showed a normal distribution in which two factors were analyzed, the double factor analysis of variance test (ANOVA) was used. We considered as a factor 1 the time exposure and as factor 2 the smoking. When it was necessary to perform multiple comparisons of means, the Bonferroni test was used. When the data did not present a normal distribution, we used the Mann-Whitney test for the group factor. For the time factor, the Kruskal-Wallis test was used, and when it was necessary to perform multiple comparisons, the Dunn test was used.

## 3 Results

### 3.1 Cell and bone mineral matrix evaluation

The histomorphometric parameters of cell and bone mineral matrix evaluation were analyzed in the control and smoking groups at 1 (C1 and S1), 3 (C3 and S3) and 6 months (C6 and S6) follow-up and included **structural parameters** (BV/TV, Tb.Th, Tb.N and Tb.Sp), **resorptive remodeling parameters** (ES/BS and Oc.S/BS) and **formative remodeling parameters** (O.Th, OS/BS and Ob.S/BS).

**Figure 3:**
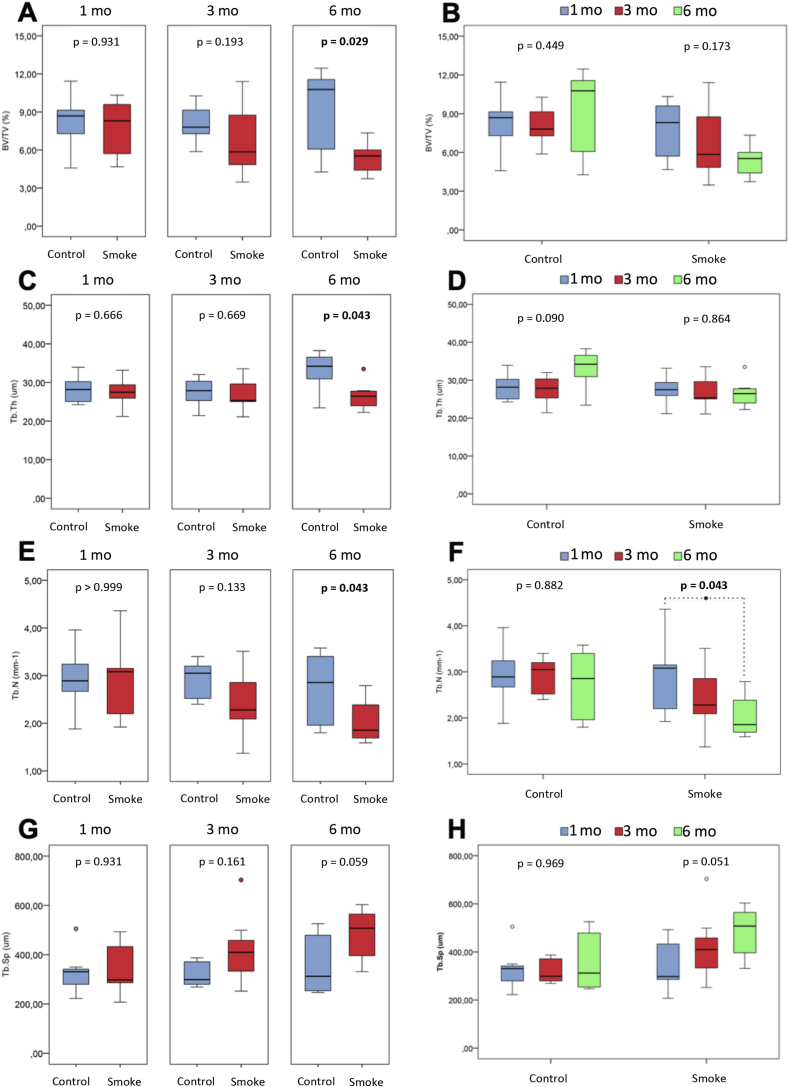
Histomorphometric assessment of structural parameters. Statistical analyses were performed with Mann-Whitney test in A, C, E and G; Kruskal-Wallis test in B, D, F and H. The data are shown as median and interquartile range IQ (25–75%). mo: month; BV/TV: relationship between trabecular bone volume and total bone volume; Tb.Th: trabecular thickness; Tb.N: trabecular number; Tb.Sp: trabecular separation. ∗Statistically significant difference present in the Dunn post-test.

The assessment of **structural parameters** (Figure 3 A-H) showed no difference between the groups with 1 and 3 months of follow-up (C1 x S1 and C3 x S3). However, there was a significant reduction in the BV/TV (p = 0.029), Tb.Th (p = 0.043) and Tb.N (p = 0.043) parameters in the groups of exposure to cigarette smoke when compared to the control groups with 6 months of follow-up (C6 x S6). There was also an increase in the Tb.Sp parameter in the exposure group compared to the control group also with 6 months of follow-up (C6 x S6), but not statistically significant (p = 0.059).

There was no difference between the control groups over time for all structural parameters (C1 x C3 x C6). However, there was a significant reduction in the parameter Tb.N (p = 0.043) between groups of exposure to cigarette smoke over time (S1 x S3 x S6), with Dunn's post-test for multiple comparisons showed that the difference occurred between S1 x S6 (p = 0.037). Likewise, we noticed a visible increase in the parameter Tb. Sp between the groups of exposure to cigarette smoke over time (S1 x S3 x S6), but not statistically significant (p = 0.051).

**Figure 4:**
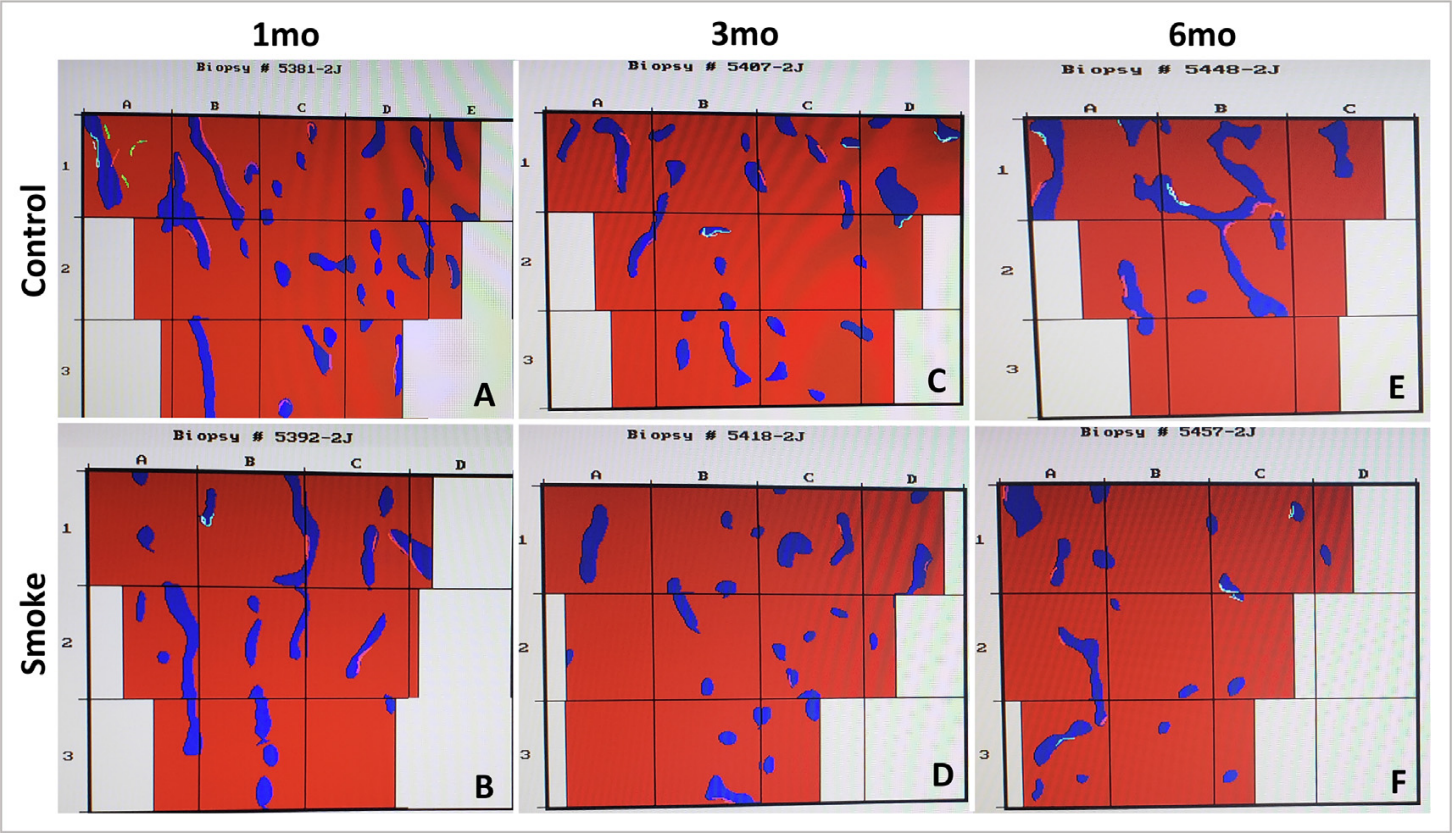
Digitalized image of the tibial metaphyseal region in red where the blue trabeculae are shown in blue. Histomorphometric samples of control and cigarette smoke groups at 1, 3 and 6 months of exposure. A, C and E: control 1, 3 and 6 months, respectively; B, D, F: smoke 1, 3 and 6 months, respectively. mo: month.

For these results, we noticed that exposure to cigarette smoke generated changes in bone structural parameters in the 6th month of follow-up, with a reduction in bone volume, trabecular thickness and trabecular number, demonstrating an evident bone loss (Figure 4). In addition, we found that this worsening was progressive over time for the trabecular number.

**Figure 5:**
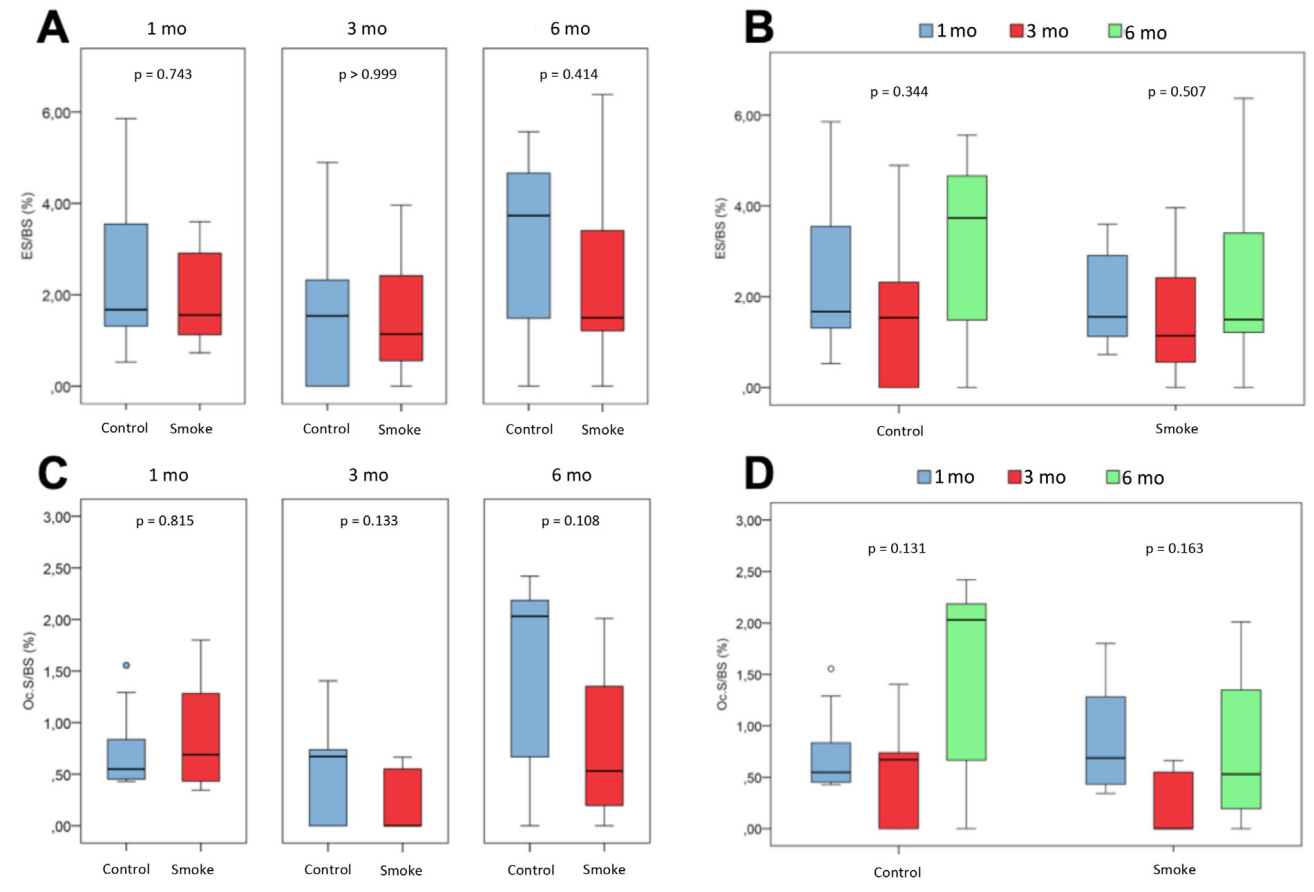
Histomorphometric assessment of resorptive remodeling parameters. Statistical analyses were performed with Mann-Whitney test in A and C; Kruskal-Wallis test in B and D. The data are shown as median and interquartile range IQ (25–75%). mo: month; ES/BS: eroded surface area; Oc.S/BS: osteoclastic surface.

The assessment of **resorptive remodeling parameters** (Figure 5 A-D) showed no difference between the experimental groups (C1 x S1, C3 x S3 and C6 x S6) for none of the two parameters analyzed (ES/BS and Oc.S/BS). Likewise, there was no difference between groups over time (C1 x C3 x C6 and S1 x S3 x S6).

**Figure 6:**
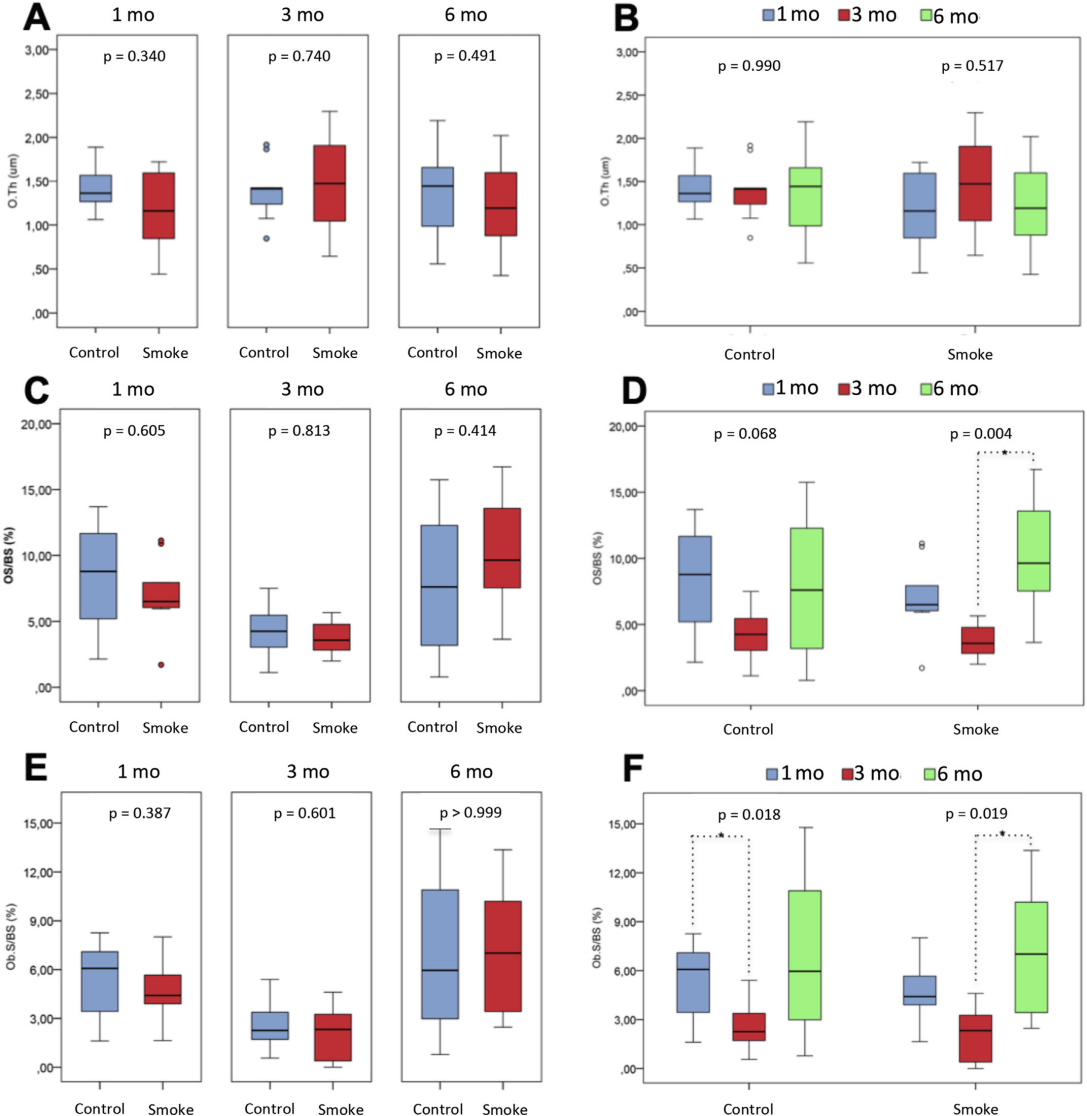
Histomorphometric assessment of formative remodeling parameters. Statistical analyses were performed with Mann-Whitney test in A, C and E; Kruskal-Wallis test in B, D and F. The data are shown as median and interquartile range IQ (25–75%). mo: month; O.Th: osteoid thickness; OS/BS: osteoid surface area; Ob.S/BS: osteoblastic surface. ∗Statistically significant difference present in the Dunn post-test.

The assessment of **formative remodeling parameters** (Figure 6 A-F) showed no difference between the experimental groups (C1 x S1, C3 x S3 and C6 x S6) for any of the three parameters analyzed (O.Th, OS/BS and Ob.s/BS). However, there was a significant increase in the OS/BS parameter (p = 0.004) between groups of exposure to cigarette smoke over time (S1 x S3 x S6), and Dunn's post-test for multiple comparisons evidenced that the difference occurred between S3 x S6 (p = 0.003). Likewise, there was a significant increase in the Ob.S/BS parameter (p = 0.019) between groups of exposure to cigarette smoke over time (S1 x S3 x S6), with Dunn's post-test for multiple comparisons it was evidenced that the difference occurred between S3 x S6 (p = 0.018).

### 3.2 Cytokines analysis

**Figure 7:**
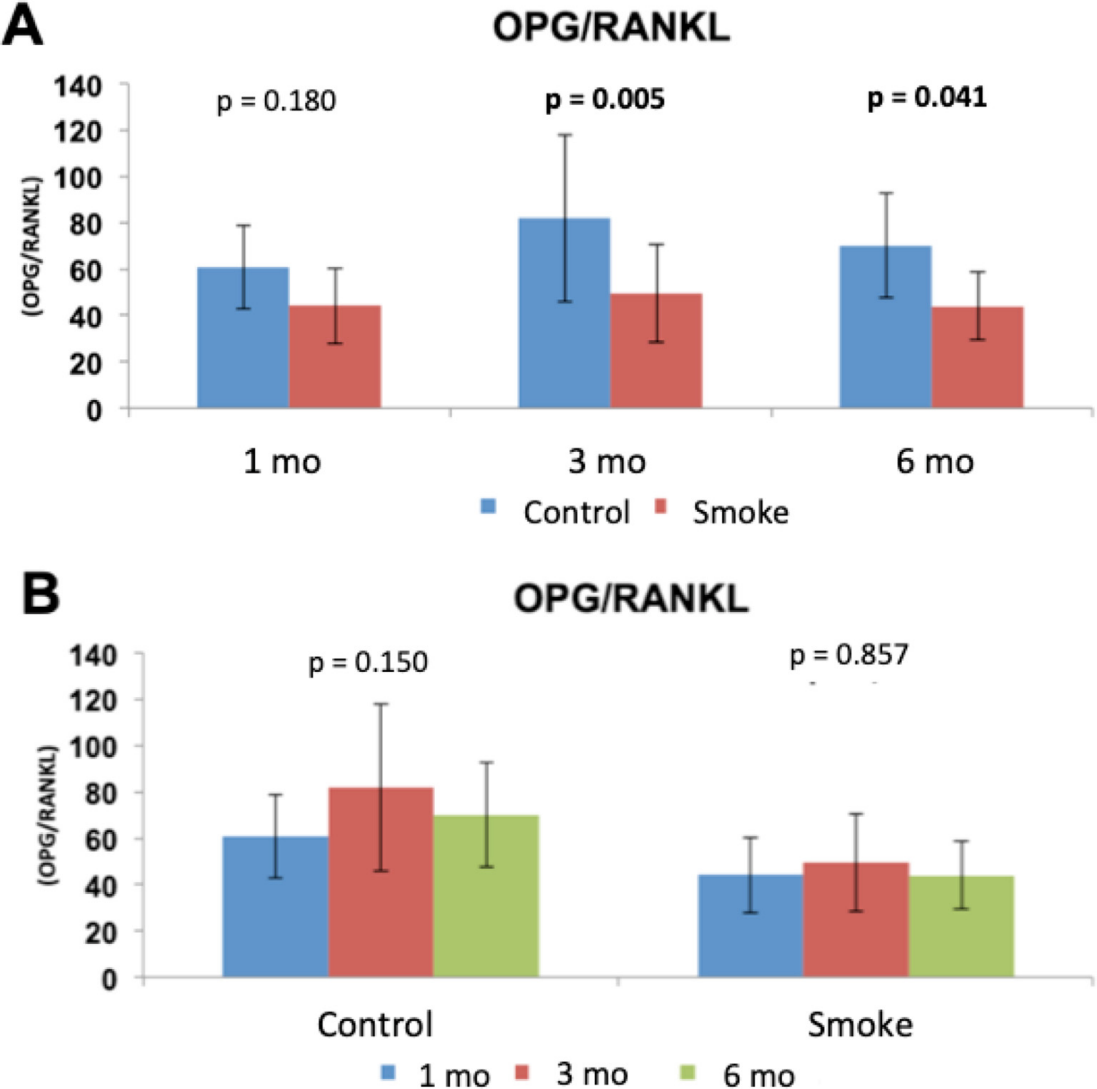
Assessment of OPG/RANKL ratio. Statistical analyses were performed with double factor analysis of variance test (ANOVA) in A and B. The data are shown as mean and standard deviation. mo: month.

The evaluation of the expression of cytokines (OPG and RANKL, described by the **OPG/RANKL** ratio) in the smoking and control groups at 1 (C1 and S1), 3 (C3 and S3) and 6 months (C6 and S6) are shown in Figure 7.

The assessment of OPG and RANKL cytokine expression showed a reduction in the **OPG/RANKL** ratio in groups exposed to cigarette smoke when compared to control groups with 3 and 6 months of follow-up (Figure 7A): C1 x S1 (p = 0.180); C3 x S3 (p = 0.005); C6 x S6 (p = 0.041). There was no significant difference among control and experimental group over time (Figure 7B): C1 x C3 x C6 (p = 0.150); S1 x S3 x S6 (p = 0.857). The results also showed that for this parameter (OPG/RANKL), the control condition and smoking are independent of time.

Therefore, exposure to cigarette smoke generated a reduction in OPG/RANKL ratio closely related to the increase in osteoclastogenic activity and bone resorption from the 3rd month on, but there was no evidence of a progressive effect over time.

### 3.3 Thiobarbituric acid reactive substances (Tbars) analysis

**Figure 8:**
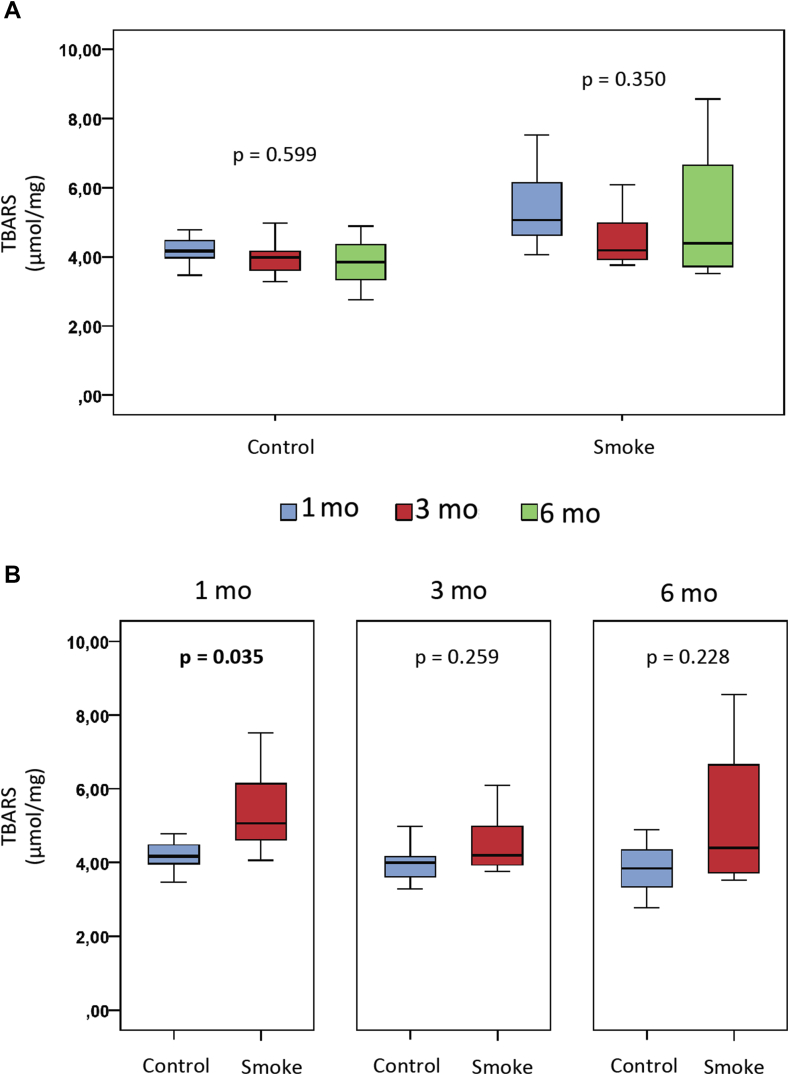
Thiobarbituric acid reactive substances (Tbars) analysis. Statistical analyses were performed with Kruskal-Wallis test in A and Mann-Whitney test in B. The data are shown as median and interquartile range IQ (25–75%). mo: month.

The evaluation of the thiobarbituric acid reactive substances (Tbars) in the smoking and control groups at 1 (C1 and S1), 3 (C3 and S3) and 6 months (C6 and S6) are shown in Figure 8.

There was no difference between the control groups or smoke groups over time (Figure 8A). However, there was a significant increase in Tbars (p = 0.035) in the group of exposure to cigarette smoke when compared to the control group with 1 month of follow-up (Figure 8B).

Therefore, exposure to cigarette smoke generated an increase in Tbars related to the increase in osteoclastogenic activity and bone resorption in the first month, but there was no evidence of a progressive effect over time.

## 4 Discussion

In this study, we showed that exposure to CS leads the increase of reactive oxygen species (ROS) previously to the increase of osteoclastogenesis-related mediators, culminating in the worsening of the bone structural parameters.

We demonstrated by the mineral matrix analysis, that there was increased osteoclastic action evidenced by the reduction in bone volume, trabecular thickness and the number of trabeculae associated after 6 months of CS exposure. Corroborating with these findings, previously our group demonstrated that mice exposed to cigarette smoke for 45 days presented worsening of some static and dynamic formative histomorphometric variables [6].

The increase in ROS production could interfere in cells activities. When there is an imbalance between ROS formation and the capability of cells to remove them, the oxidative stress mechanism will occur, affecting cellular structures and activities [24].

In the 90's, the first studies that described the relationship between oxygen-derived free radicals and the osteoclastic formation and activation process appeared [25]. ROS production is particularly involved in mineral tissue homeostasis and contributes mostly to bone remodelling by promoting bone resorption [16]. Oxidative stress is also related with reduced bone mineral density and osteoporosis by *in vitro* and animal studies [26, 27]. Decreased bone mineral density (BMD) was shown to be associated with higher oxidative stress index values and total plasma oxidant status in osteoporotic patients [28].

ROS has also been found to influence several different signaling pathways and are produced following RANKL stimulation of the undifferentiated cell [15]. In human osteoblast-like cells, increased intracelular ROS levels were shown to stimulate RANKL mRNA and protein expression [29]. In our study, the increase in Tbars has already occurred in the first month of exposure to cigarette smoke prior to the increase in RANKL as would be expected, without, however, having a progressive effect identified over time.

Temporally, we noticed that there is initially an increase in ROS production, followed by an increase in RANKL (with greater osteoclastic activation) and a consequent bone structural deterioration. These three events occur simultaneously in the bone tissue, but it showed more intensity at 1, 3 and 6 months respectively, which could explain a chronological sequence.

It has been described in literature the association of long-term exposure to cigarette smoke and increased reabsorptive function of osteoclasts [6]. In a clinical study, Kulak et al. [30], also showed in postmenopausal women with COPD, a worsening of the bone structural parameters when evaluated by microtomography and histomorphometry.

Although we were unable to capture an increase in osteoclastic cells on the surface of the trabecula, it is possible that these cells were more active, since there was an evident structural bone deterioration associated with exposure to cigarette smoke. It is important to note that there are some limitations in using histomorphometry analysis in small rodents. Since this analysis was developed to describe structural changes in bone of humans, and considering the anatomical differences in bone structure of rodents, it is necessary severe alterations in mice to be detected [31].

Increased RANKL/OPG ratio (or decreased OPG/RANKL ratio) is also related to local osteoclastogenic activity and bone resorption in several pathologies such as periodontitis [14], rheumatoid arthritis [32], multiple myeloma [33], osteoporosis [34] and COPD [35, 36]. In our study, we evidenced a reduction in the OPG/RANKL ratio from the 3rd month onwards, with no progressive effect identified over time. The structural worsening attested by histomorphometry analysis were found to be subsequent to these events, which would be expected, since the reabsorptive stimulus must precede the resorption itself.

## 5 Conclusion

Our findings showed the sequence of events that leads the impairment in bone turn over and changes in bone structure related to exposure to cigarette smoke. We initially showed an increase in Tbars (oxidative stress), followed by an increase in RANKL (cytokines associated with osteoclastogenesis) and, finally, the structural bone change revealed by histomorphometry.

## Declarations

### Author contribution statement

Jader Joel Machado Junqueira, Fernanda Degobbi Tenorio Quirino dos Santos Lopes: Conceived and designed the experiments; Performed the experiments; Analyzed and interpreted the data; Contributed reagents, materials, analysis tools or data; Wrote the paper.

Juliana Dias Lourenço: Performed the experiments; Contributed reagents, materials, analysis tools or data; Wrote the paper.

Kaique Rodrigues da Silva: Performed the experiments; Contributed reagents, materials, analysis tools or data.

Vanda Jorgetti, Rodolfo de Paula Vieira, Amanda Aparecida de Araujo, Kátia De Angelis: Performed the experiments; Analyzed and interpreted the data; Contributed reagents, materials, analysis tools or data.

Aristides Tadeu Correia: Analyzed and interpreted the data.

Luan Henrique Vasconcelos Alves: Wrote the paper.

Iolanda de Fátima Lopes Calvo Tibério, Alexandre Póvoa Barbosa: Conceived and designed the experiments; Analyzed and interpreted the data.

### Funding statement

This study was supported by 10.13039/501100001807Fundação de Amparo à Pesquisa do Estado de São Paulo (FAPESP).

### Data availability statement

Data will be made available on request.

### Declaration of interests statement

The authors declare no conflict of interest.

### Additional information

No additional information is available for this paper.

## References

[1] Talhout R., Schulz T., Florek E., van Benthem J., Wester P., Opperhuizen A. (2011). Hazardous compounds in tobacco smoke. Int. J. Environ. Res. Publ. Health.

[2] Ezzati M., Lopez A.D. (2003). Estimates of global mortality attributable to smoking in 2000. Lancet.

[3] Sasaki M., Chubachi S., Kameyama N., Sato M., Haraguchi M., Miyazaki M., Takahashi S., Nakano T., Kuroda Y., Betsuyaku T., Matsuo K. (2018). Effects of long-term cigarette smoke exposure on bone metabolism, structure, and quality in a mouse model of emphysema. PLoS One.

[4] Pearson R.G., Clement R.G.E., Edwards K.L., Scammell B.E. (2016). Do smokers have greater risk of delayed and non-union after fracture, osteotomy and arthrodesis? A systematic review with meta-analysis. BMJ Open.

[5] Daniel H.W. (1976). Osteoporosis of the slender smoker. Vertebral compression fractures and loss of metacarpal cortex in relation to postmenopausal cigarette smoking and lack of obesity. Arch. Intern. Med..

[6] Barbosa A.P., Lourenço J.D., Junqueira J.J.M., Larissa Emidio de França S., Martins J.S., Oliveira Junior M.C., Begalli I., Velosa A.P.P., Olivo C.R., Bastos T.B., Jorgetti V., Rodolfo de Paula V., Teodoro W.R., Lopes F.D.T.Q.S. (2020). The deleterious effects of smoking in bone mineralization and fibrillar matrix composition. Life Sci..

[7] Broulik P., Jaráb J. (1993). The effect of chronic nicotine administration on bone mineral content in mice. Horm. Metab. Res..

[8] Henemyre C.L., Escalas D.K., Hokett S.D., Cuenin M.F., Pavão M., Parker M.H., Brewer P.D., Chuang A.H. (2003). Nicotine stimulates osteoclast resorption in a porcine marrow cell model. J. Periodontol..

[9] Kamer A.R., El-Ghorab N., Marzec N., Margarone J.E., Dziak R. (2006). Nicotine induced proliferation and cytokine release in osteoblastic cells. Int. J. Mol. Med..

[10] Mescher A. (2016).

[11] Siddiqui J.A., Partridge N.C. (2016). Physiological bone remodeling: systemic regulation and growth factor involvement. Physiol..

[12] Saha H., Mukherjee B., Bindhani B., Ray M.R. (2016). Changes in RANKL and osteoprotegerin expression after chronic exposure to indoor air pollution as a result of cooking with biomass fuel. J. Appl. Toxicol..

[13] Castrogiovanni P., Trovato F.M., Szychlinska M.A., Nsir H., Imbesi R., Musumeci G. (2016). The importance of physical activity in osteoporosis. From the molecular pathways to the clinical evidence. Histol. Histopathol..

[14] Lappin D.F., Sherrabeh S., Jenkins W.M.M., Macpherson L.M.D. (2007). Effect of smoking on serum RANKL and OPG in sex, age and clinically matched supportive-therapy periodontitis patients. J. Clin. Periodontol..

[15] Callaway D.A., Jiang J.X. (2015). Reactive oxygen species and oxidative stress in osteoclastogenesis, skeletal aging and bone diseases. J. Bone Miner. Metabol..

[16] Wauquier F., Leotoing L., Coxam V., Guicheux J., Wittrant Y. (2009). Oxidative stress in bone remodelling and disease. Trends Mol. Med..

[17] Lee N.K., Choi Y.G., Baik J.Y., Han S.Y., Jeong D.W., Bae Y.S., Kim N., Lee S.Y. (2005). A crucial role for reactive oxygen species in RANKL-induced osteoclast differentiation. Blood.

[18] (2011). Committee for the Update of the Guide for the Care and Use of Laboratory Animals..

[19] Toledo A.C., Magalhaes R.M., Hizume D.C., Vieira R.P., Biselli P.J.C., Moriya H.T., Mauad T., Lopes F.D.T.Q.S., Martins M.A. (2012). Aerobic exercise attenuates pulmonary injury induced by exposure to cigarette smoke. Eur. Respir. J..

[20] J D. (1974). A simplified method for the preparation of methyl methacrylate embedding medium for undecalcified bone. Med. Lab. Technol..

[21] Dempster D.W., Compston J.E., Drezner M.K., Glorieux F.H., Kanis A.J., Malluche H., Meunier P.J., Ott S.M., Recker R.R., Parfitt M.A. (2013). Standardized nomenclature, symbols, and units for bone histomorphometry: a 2012 update of the report of the ASBMR histomorphometry nomenclature committee. J. Bone Miner. Res..

[22] Esterbauer H., Cheeseman K.H. (1990). Determination of aldehydic lipid peroxidation products: malonaldehyde and 4-hydroxynonenal. Methods Enzymol..

[23] Lowry O.H., Rosebrough N.J., Farr A.L., Randall R.J. (1951). Protein measurement with the Folin phenol reagent. J. Biol. Chem..

[24] Van Der Vaart H., Postma D.S., Timens W., Ten Hacken N.H.T. (2004). Acute effects of cigarette smoke on inflammation and oxidative stress: a review. Thorax.

[25] Garrett I.R., Boyce B.F., Oreffo R.O.C., Bonewald L., Poser J., Mundy G.R. (1990). Oxygen-derived free radicals stimulate osteoclastic bone resorption in rodent bone in vitro and in vivo. J. Clin. Invest..

[26] Bai X.C., Lu D., Bai J., Zheng H., Ke Z.Y., Li X.M., Luoa S. (2004). Oxidative stress inhibits osteoblastic differentiation of bone cells by ERK and NF-kappaB. Biochem. Biophys. Res. Commun..

[27] Lean J.M., Jagger C.J., Kirstein B., Fuller K., Chambers T.J. (2005). Hydrogen peroxide is essential for estrogen-deficiency bone loss and osteoclast formation. Endocrinology.

[28] Altindag O., Erel O., Soran N., Celik H., Selek S. (2008). Total oxidative/anti-oxidative status and relation to bone mineral density in osteoporosis. Rheumatol. Int..

[29] Bai X.C., Lu D., Liu A.L., Zhang Z.M., Li X.M., Zou Z.P., Sen Zeng W., Cheng B.L., Luo S.Q. (2005). Reactive oxygen species stimulates receptor activator of NF-κB ligand expression in osteoblast. J. Biol. Chem..

[30] Kulak C.A.M., Borba V.C., Jorgetti V., Dos Reis L.M., Liu X.S., Kimmel D.B., Kulak J., Rabelo L.M., Zhou H., Guo X.E., Bilezikian J.P., Boguszewski C.L., Dempster D.W. (2010). Skeletal microstructural abnormalities in postmenopausal women with chronic obstructive pulmonary disease. J. Bone Miner. Res..

[31] Recker R.R., Kimmel D.B., Dempster D., Weinstein R.S., Wronski T.J., Burr D.B. (2011). Issues in modern bone histomorphometry. Bone.

[32] Xu S., Wang Y., Lu J., Xu J. (2012). Osteoprotegerin and RANKL in the pathogenesis of rheumatoid arthritis-induced osteoporosis. Rheumatol. Int..

[33] Terpos E., Szydlo R., Apperley J.F., Hatjiharissi E., Politou M., Meletis J., Viniou N., Yataganas X., Goldman J.M., Rahemtulla A. (2003). Soluble receptor activator of nuclear factor κB ligand-osteoprotegerin ratio predicts survival in multiple myeloma: proposal for a novel prognostic index. Blood.

[34] Zhang P.F., Pan L., Luo Z.Y., Zhao H.J., Cai S.X. (2013). Interrelationship of circulating matrix metalloproteinase-9, TNF-α, and OPG/RANK/RANKL systems in COPD patients with osteoporosis. COPD.

[35] Bai P., Sun Y., Jin J., Hou J., Li R., Zhang Q., Wang Y. (2011). Disturbance of the OPG/RANK/RANKL pathway and systemic inflammation in COPD patients with emphysema and osteoporosis. Respir. Res..

[36] Ugay L., Kochetkova E., Nevzorova V., Maistrovskaia Y. (2016). Role of osteoprotegerin and receptor activator of nuclear factor-κB ligand in bone loss related to advanced chronic obstructive pulmonary disease. Chin. Med. J..

